# Favourable Experience with M-Mode Sonography in the Diagnosis of Pneumothorax in Two Patients with Thoracic Subcutaneous Emphysema

**DOI:** 10.1155/2014/906127

**Published:** 2014-11-13

**Authors:** T. Berlet, R. Etter

**Affiliations:** Department of Intensive Care Medicine, Inselspital, Bern University Hospital and University of Bern, 3010 Bern, Switzerland

## Abstract

*Introduction*. Thoracic subcutaneous emphysema may be caused by various pathologies. In mechanically ventilated patients, timely diagnostic workup is particularly important, as the presence of subcutaneous surgical emphysema may indicate pneumothorax, representing a risk factor for the development of life threatening tension pneumothorax. Thoracic ultrasound is of proven value for the detection of pneumothorax but has so far been considered of little value in the context of subcutaeneous emphysema, due to poor visibility of anatomic structures. *Case Presentation*. We present the successful use of diagnostic M-mode sonography in two mechanically ventilated patients who developed thoracic subcutaneous emphysema. In both cases B-mode sonography was inconclusive. *Conclusion*. M-mode sonography may be more sensitive than B-mode sonography in the detection of lung sliding and could become a useful diagnostic tool when pneumothorax needs to be ruled in or ruled out and visibility of the pleura is limited.

## 1. Introduction

Thoracic subcutaneous emphysema may be caused by injuries to the airways, pharynx, oesophagus, abdomen or retroperitoneal space, severe soft-tissue infections, or pneumothorax [1]. In mechanically ventilated patients, the presence of subcutaneous surgical emphysema is always a cause for concern as it may indicate pneumothorax along with the risk for the development of life threatening tension pneumothorax. Physical examination and portable plain chest X-ray are both frequently unreliable in this scenario. CT-scanning, while highly sensitive and specific, is usually not feasible in this situation [2]. Thoracic ultrasound is of proven value for the diagnosis of pneumothorax [3]. However caution is warranted in the setting of thoracic subcutaneous emphysema due to poor visibility of anatomic structures and particularly of the pleura [4]. We report our favourable experience with the use of M-mode sonography to rule in and rule out pneumothorax in two mechanically ventilated patients who developed subcutaneous emphysema. In both cases a portable Sonosite M-Turbo ultrasound unit (SonoSite Inc., Bothell, WA, USA) in connection with L-38 broadband linear array 5–10 MHz transducer was used.

## 2. Case Reports

**Case 1 .**

**Figure 1:**
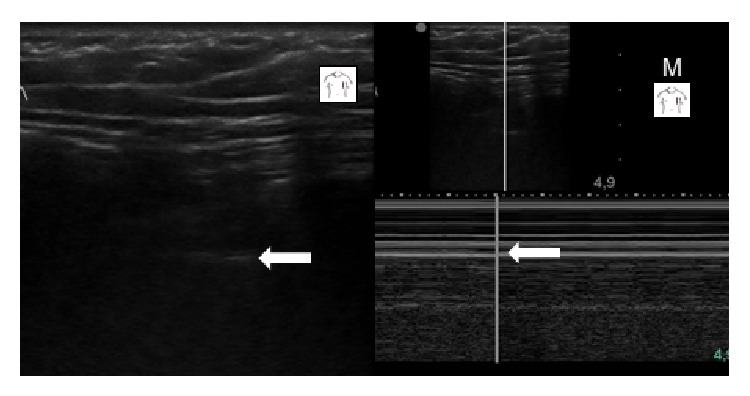
Case 1: B-mode and M-mode sonography using a linear transducer positioned in the mid-clavicular line at the 3rd left intercostal space. Arrows indicate the pleural lines (B: B-mode, M: M-mode).

**Figure 2:**
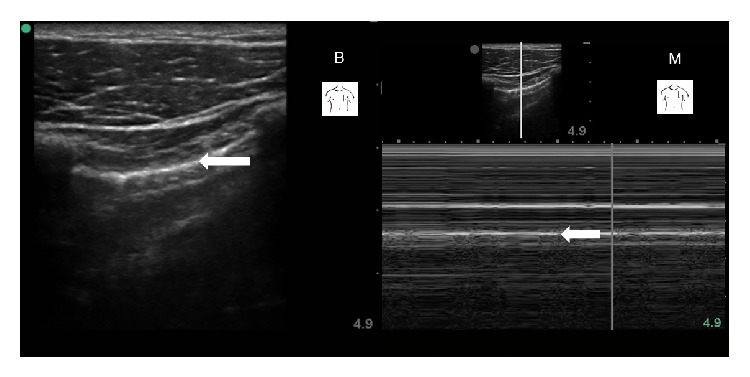
Healthy volunteer for comparison: B-mode and M-mode sonography using a linear transducer positioned in the mid-clavicular line at the 3rd intercostal space. Arrows indicate the pleural lines (B: B-mode, M: M-mode).

A 54-year-old male was admitted to our ICU following emergency coronary bypass artery grafting for a myocardial infarct complicated by acute heart failure. He remained on mechanical ventilation for 8 days. Following reinsertion of a central venous catheter into the right subclavian vein on day 6, subcutaneous emphysema developed; it was located predominantly at the front of the patient's left hemithorax. Thoracic ultrasonography was performed to rule out pneumothorax as a possible cause. In B-mode sonography, the pleural lines were barely visible bilaterally, despite the operator's effort to displace the subcutaneous gas by applying pressure to the soft tissue with the transducer. Lung sliding could not be identified with confidence. Neither comet-tail artifacts nor lung point nor lung pulse could be seen (see additional electronic content “Case 1” available online at http://dx.doi.org/10.1155/2014/906127). In M-mode sonography, however, signs of lung-sliding (“sea-shore sign”) were easily visualised. In fact, the ultrasound pattern in M-mode sonography was almost as distinct as in a healthy volunteer (Figures 1 and 2). Emphysema subsided in the subsequent days without the need for an intervention.

**Case 2 .**

**Figure 3:**
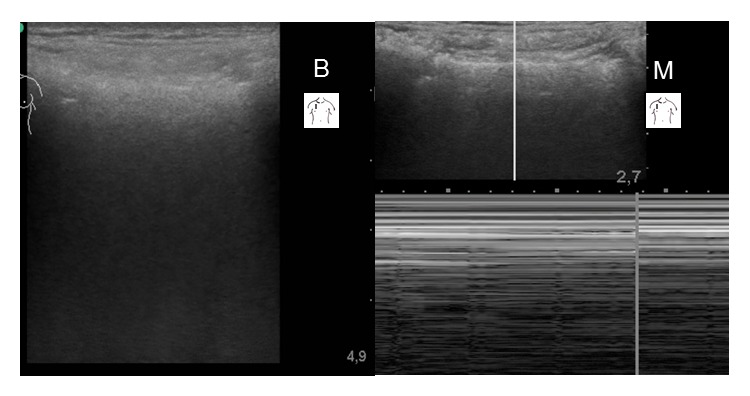
Case 2: B-mode and M-mode sonography using a linear transducer positioned in the mid-clavicular line at the 3rd right intercostal space (B: B-mode, M: M-mode).

A 73-year-old male who underwent chemotherapy for chronic lymphocytic leukaemia was admitted to our ICU with hypoxaemic respiratory failure caused by hospital acquired pneumonia. Invasive mechanical ventilation had to be initiated on day two. Acute respiratory distress syndrome (ARDS) ensued. On day 10 the patient developed right sided thoracic subcutaneous emphysema, accompanied by signs and symptoms of worsening respiratory function. Thoracic ultrasonography was performed to rule in or rule out pneumothorax as a possible cause. Similar to Case 1, in B-mode scanning chest wall structures and pleural lines were barely visible; the presence or absence of lung sliding could not be confirmed (see additional electronic content “Case 2”). In M-mode sonography the absence of lung sliding (“barcode signs”) was detected (Figure 3). Pneumothorax was confirmed during chest tube insertion. Following the intervention the patient's respiratory condition improved rapidly.

## 3. Discussion

Our experience confirms the limited ability of B-mode sonography to confirm or exclude pneumothorax when thoracic subcutaneous emphysema is present [4]. While both M-mode and Colour Doppler sonography have been described as alternative modes in the examination of the pleura, no suggestion has been made so far that these modes might carry superior diagnostic capabilities in terms of sensitivity or specificity [5–7]. When we applied M-mode sonography after localising the intercostal spaces with B-mode sonography we were able to correctly diagnose the absence and strongly suspect the presence of pneumothorax in two very challenging clinical situations without difficulties. Our explanation for this phenomenon is twofold.

Firstly, the single ultrasound beam of M-mode sonography allows the operator to focus on a suitable acoustic window, whereas the large acoustic footprint of B-mode sonography causes the field of view to be “crowded” by reverberation artifacts originating from subcutaneous gas bubbles. Secondly, the high temporal resolution of M-mode sonography as compared to 2D techniques facilitates the recognition of even subtle motion artifacts caused by lung sliding [8]. A possible disadvantage of M-mode sonography could be the difficulty to accurately localise the pleural interface. This would be mainly due to the presence of multiple hyperechoic lines along the path of the ultrasound beam, each representing an interface between two tissue layers with different acoustic properties.

## 4. Conclusion

We conclude that M-mode sonography is more sensitive than B-mode sonography in detecting even subtle motion artifacts caused by lung sliding, may be a useful diagnostic tool for pneumothorax when visibility of the pleura is limited, and merits systematic evaluation regarding its overall diagnostic accuracy.

## Supplementary Material

Case1: Real time B-mode sonography using a linear transducer positioned in the mid-clavicular line at the 3rd intercostal space.
Case 2: Real time B-mode sonography using a linear transducer positioned in the mid-clavicular line at the 3rd intercostal space
